# Supplementary material for: The C-terminal domain of Hsp70 is responsible for paralog-specific regulation of ribonucleotide reductase
Source: PLoS Genet. 2022 Apr 13;18(4):e1010079. doi: 10.1371/journal.pgen.1010079 (PMC9037926; doi:10.1371/journal.pgen.1010079)
Supplement: S1 File — Table A. Yeast Strains Used in This Study. Table B. Plasmids Used in This Study. Table C. RT PCR Primers Used in This Study. (DOCX) [file pgen.1010079.s001.docx]

**Table A. Yeast Strains Used in This Study**

| **Strain** | **Genotype** | **Reference/Source** |
| --- | --- | --- |
| yAT414 | MATa (MH272) *ssa1∆::trp1 ssa2::HisG ssa3::HisG ssa4::HisG (ssa1-4)* [YCPlac33 SSA1] | [1] |
| yAT423 | MATa (MH272) *ssa1∆::trp1 ssa2::HisG ssa3::HisG ssa4::HisG (ssa1-4)* [YCPlac33 SSA1]):: RNR1-HA-HIS3MX6 | This study |
| yAT424 | MATa (MH272) *ssa1∆::trp1 ssa2::HisG ssa3::HisG ssa4::HisG (ssa1-4)* [YCPlac33 SSA1]):: RNR2-HA-HIS3MX6 | This study |
| yAT425 | MATa (MH272) *ssa1∆::trp1 ssa2::HisG ssa3::HisG ssa4::HisG (ssa1-4)* [YCPlac33 SSA1]):: RNR4-HA-HIS3MX6 | This study |
| yAT307 | MATa (PJ69-4a) pOAD *RNR1* | [2] |
| yAT305 | MATa (PJ69-4a) pOAD *RNR2* | [2] |
| yAT301 | MATa (PJ69-4a) | [2] |

**Table B. Plasmids Used in This Study**

| **Plasmid** | **Description** | **Reference/Source** |
| --- | --- | --- |
| pAT625 | pRS315P_SSA2_-SSA1(LEU2) | [3] |
| pAT626 | pRS315P_SSA2_-SSA2(LEU2) | [3] |
| pAT627 | pRS315P_SSA2_-SSA3(LEU2) | [3] |
| pAT628 | pRS315P_SSA2_-SSA4(LEU2) | [3] |
| pAT659 | pRS315P_SSA2_-SSA24(LEU2) | [4] |
| pAT660 | pRS315P_SSA2_-SSA42(LEU2) | [4] |
| pAT592 | RNR3 promoter-lacZ | [5] |
| pAT289 | p*BD-SSA1* | [6] |
| pAT293 | p*BD-SSA2* | [6] |
| pAT283 | p*BD-SSA3* | [6] |
| pAT294 | p*BD-SSA4* | [6] |
| pAT295 | p*BD-SSA4* | [6] |
| pAT739 | pOAD-RNR4 | This Study |
| pAT716 | pBG1085-GAL1 promoter-ZZ-HA-RNR1 | Dharmacon |
| pAT717 | pBG1085-GAL1 promoter-ZZ-HA-RNR2 | Dharmacon |
| pAT718 | pBG1085-GAL1 promoter-ZZ-HA-RNR4 | Dharmacon |
| pAT705 | pFA6a-HA-HIS3MX6 | [7] |

**Table C. RT PCR Primers Used in This Study**

| **Gene** | **Primers** |
| --- | --- |
| RNR1 forward | 5′-GTGTTCAAGGTCTCGCTGAC-3′ |
| RNR1 forward | 5′-CGTATGGACCGTCCTTCTGA-3′ |
| RNR2 forward | 5′-CCTAAAGAGACCCCTTCCAAAG-3′ |
| RNR2 reverse | 5′-GCCTTGTGATTTTCAGCGTC-3′ |
| RNR3 forward | 5′-GCCTCCGCTGCTATTCAA-3′ |
| RNR3 reverse | 5′- CAGATGCCGCCTTTTGTT-3′ |
| RNR4 forward | 5′-CATAAGGCTGCTTTCATCGAG-3′ |
| RNR4 reverse | 5′-CTGTTGGCCATTGCTAAACC-3′ |
| ACT1 forward | 5′-GTATGTGTAAAGCCGGTTTTG-3′ |
| ACT1 reverse | 5′-CATGATACCTTGGTGTCTTGG-3′ |

**References**

1. Jaiswal H, Conz C, Otto H, Wolfle T, Fitzke E, Mayer MP, et al. The chaperone network connected to human ribosome-associated complex. Mol Cell Biol. 2011;31(6):1160-73. Epub 2011/01/20. doi: 10.1128/MCB.00986-10. PubMed PMID: 21245388; PubMed Central PMCID: PMCPMC3067906.

2. Uetz P, Giot L, Cagney G, Mansfield TA, Judson RS, Knight JR, et al. A comprehensive analysis of protein-protein interactions in Saccharomyces cerevisiae. Nature. 2000;403(6770):623-7. Epub 2000/02/25. doi: 10.1038/35001009. PubMed PMID: 10688190.

3. Sharma D, Masison DC. Functionally redundant isoforms of a yeast Hsp70 chaperone subfamily have different antiprion effects. Genetics. 2008;179(3):1301-11. Epub 2008/06/20. doi: 10.1534/genetics.108.089458. PubMed PMID: 18562668; PubMed Central PMCID: PMCPMC2475734.

4. Gaur D, Singh P, Guleria J, Gupta A, Kaur S, Sharma D. The Yeast Hsp70 Cochaperone Ydj1 Regulates Functional Distinction of Ssa Hsp70s in the Hsp90 Chaperoning Pathway. Genetics. 2020;215(3):683-98. Epub 2020/04/18. doi: 10.1534/genetics.120.303190. PubMed PMID: 32299842; PubMed Central PMCID: PMCPMC7337085.

5. Zhou Z, Elledge SJ. Isolation of crt mutants constitutive for transcription of the DNA damage inducible gene RNR3 in Saccharomyces cerevisiae. Genetics. 1992;131(4):851-66. Epub 1992/08/01. doi: 10.1093/genetics/131.4.851. PubMed PMID: 1516817; PubMed Central PMCID: PMCPMC1205097.

6. Wegele H, Haslbeck M, Reinstein J, Buchner J. Sti1 is a novel activator of the Ssa proteins. J Biol Chem. 2003;278(28):25970-6. Epub 2003/04/30. doi: 10.1074/jbc.M301548200. PubMed PMID: 12716905.

7. Longtine MS, McKenzie A, 3rd, Demarini DJ, Shah NG, Wach A, Brachat A, et al. Additional modules for versatile and economical PCR-based gene deletion and modification in Saccharomyces cerevisiae. Yeast. 1998;14(10):953-61. Epub 1998/08/26. doi: 10.1002/(SICI)1097-0061(199807)14:10<953::AID-YEA293>3.0.CO;2-U. PubMed PMID: 9717241.
